# Separate and combined effects of individual and neighbourhood socio-economic disadvantage on health-related lifestyle risk factors: a multilevel analysis

**DOI:** 10.1093/ije/dyab079

**Published:** 2021-04-24

**Authors:** Yinjie Zhu, Ming-Jie Duan, Ineke J Riphagen, Isidor Minovic, Jochen O Mierau, Juan-Jesus Carrero, Stephan J L Bakker, Gerjan J Navis, Louise H Dekker

**Affiliations:** 1 Department of Internal Medicine, Division of Nephrology, University Medical Centre Groningen, Groningen, The Netherlands; 2 Department of Laboratory Medicine, University Medical Centre Groningen, Groningen, The Netherlands; 3 Faculty of Economics and Business, University of Groningen, The Netherlands; 4 Aletta Jacobs School of Public Health, University of Groningen, The Netherlands; 5 Department of Medical Epidemiology and Biostatistics, Karolinska Institutet, Stockholm, Sweden

**Keywords:** Socio-economic disadvantage, neighbourhood, lifestyle

## Abstract

**Background:**

Socio-economic disadvantage at both individual and neighbourhood levels has been found to be associated with single lifestyle risk factors. However, it is unknown to what extent their combined effects contribute to a broad lifestyle profile. We aimed to (i) investigate the associations of individual socio-economic disadvantage (ISED) and neighbourhood socio-economic disadvantage (NSED) in relation to an extended score of health-related lifestyle risk factors (lifestyle risk index); and to (ii) investigate whether NSED modified the association between ISED and the lifestyle risk index.

**Methods:**

Of 77 244 participants [median age (IQR): 46 (40–53) years] from the Lifelines cohort study in the northern Netherlands, we calculated a lifestyle risk index by scoring the lifestyle risk factors including smoking status, alcohol consumption, diet quality, physical activity, TV-watching time and sleep time. A higher lifestyle risk index was indicative of an unhealthier lifestyle. Composite scores of ISED and NSED based on a variety of socio-economic indicators were calculated separately. Linear mixed-effect models were used to examine the association of ISED and NSED with the lifestyle risk index and to investigate whether NSED modified the association between ISED and the lifestyle risk index by including an interaction term between ISED and NSED.

**Results:**

Both ISED and NSED were associated with an unhealthier lifestyle, because ISED and NSED were both positively associated with the lifestyle risk index {highest quartile [Q4] ISED beta-coefficient [95% confidence interval (CI)]: 0.64 [0.62–0.66], *P* < 0.001; highest quintile [Q5] NSED beta-coefficient [95% CI]: 0.17 [0.14–0.21], *P* < 0.001} after adjustment for age, sex and body mass index. In addition, a positive interaction was found between NSED and ISED on the lifestyle risk index (beta-coefficient 0.016, 95% CI: 0.011–0.021, *P*_interaction_ < 0.001), which indicated that NSED modified the association between ISED and the lifestyle risk index; i.e. the gradient of the associations across all ISED quartiles (Q4 vs Q1) was steeper among participants residing in the most disadvantaged neighbourhoods compared with those who resided in the less disadvantaged neighbourhoods.

**Conclusions:**

Our findings suggest that public health initiatives addressing lifestyle-related socio-economic health differences should not only target individuals, but also consider neighbourhood factors.

Key Messages

- Both individual and neighbourhood socio-economic disadvantages were associated with practising a worse lifestyle.
- Individual socio-economic disadvantage affected one’s lifestyle disproportionately across different neighbourhood-disadvantage levels.
- Public health initiatives should consider neighbourhood socio-economic status while targeting socio-economically disadvantaged individuals.

## Introduction

Lifestyle risk factors are key to the prevention of non-communicable diseases. Abundant epidemiological studies have demonstrated that socio-economic differences bear a considerable impact on lifestyle risk factors;^1^^,^^2^ i.e. individuals who are more socio-economically disadvantaged are more likely to have an unhealthy lifestyle (e.g. poor diet, smoking, less physical activity).^3–7^ However, variations within individual socio-economic strata remain. Meanwhile, studies have also suggested that neighbourhood socio-economic disadvantage (NSED), as an important contextual factor, had an independent effect on individual-level lifestyle risk factors.^8–18^ More insights are needed into the socio-economic disadvantage from different ecological levels at the same time to better understand the mechanisms behind socio-economically patterned lifestyle and health inequalities. Studies on both smoking and drinking habits have suggested an interaction between individual socio-economic disadvantage (ISED) and NSED,^4^^,^^19^ showing that NSED had disproportionate effects across different ISED strata on lifestyle behaviours. More precisely, the impact of NSED has been found to be greater for those who were more socio-economically disadvantaged.^20^^,^^21^ It has been suggested that less-socio-economically disadvantaged individuals may be protected by their individual resources from NSED, whereas more-socio-economically disadvantaged individuals may be more dependent on neighbourhood resources.^22^ However, those previous studies only examined single and traditional lifestyle risk factors, whereas a broader range of a combination of lifestyle factors, including emerging lifestyle factors, has rarely been studied in this context for their relationships with the combined effects of ISED and NSED.^23–25^

To our knowledge, it was still not clear whether NSED modifies the effect of ISED on a broader lifestyle risk profile. Therefore, this study investigated (i) the separate and combined effects of ISED and NSED on a combination of health-related lifestyle risk factors (lifestyle risk index); and (ii) whether NSED modifies the association between ISED and the lifestyle risk index.

## Methods

### Study design and participants

The Lifelines cohort study is a multidisciplinary prospective population-based cohort study that applies in a unique three-generation design the health and health-related behaviours of 167 729 persons living in the Netherlands. It employs a broad range of investigative procedures in assessing the biomedical, socio-demographic, behavioural, physical and psychological factors that contribute to the health and disease of the general population, with a special focus on multi-morbidity and complex genetics. Before study entry, a signed informed consent form was obtained from each participant. Adult participants (≥18 years old) were asked to complete several self-administered questionnaires regarding various aspects, including demographics, socio-economic status and lifestyle. A detailed description of the Lifelines cohort study can be found elsewhere.^26^^,^^27^ For the current study, 77 244 participants from the Lifelines cohort aged between 31 and 69 years who had available and reliable data on demographics, NSED, ISED and lifestyle were selected in the analysis (Supplementary Figure S1, available as Supplementary data at *IJE* online). The Lifelines study is conducted according to the principles of the Declaration of Helsinki and approved by the Medical Ethics Committee of the University Medical Centre Groningen, The Netherlands.

### NSED and ISED

A neighbourhood socio-economic disadvantage score was derived from principal component analysis (PCA) to summarize three NSED indicators. These indicators included: percentage of the population with the highest 20% income, percentage of the population with the lowest 20% income and percentage of the population receiving social benefits. NSED data were derived from the Neighbourhood Statistics (year 2011) of Statistics Netherlands (CBS), which is in accordance with the Lifelines baseline assessment. Neighbourhoods with <10 inhabitants were excluded and each neighbourhood was identified by a unique neighbourhood code. Component 1 from PCA analysis was selected to form the NSED score (Supplementary Description, available as Supplementary data at *IJE* online). The derived NSED score was subsequently divided into quintiles, with higher quintiles indicating more disadvantaged neighbourhoods.

An individual socio-economic disadvantage score was determined using factor analysis of mixed data (FAMD) to summarize four ISED variables at baseline: education, income, status of social benefits and unemployment status. Since information on education and income was not available for all participants, multiple imputation [education (0.31%) and income (14.7%)] was conducted with FAMD analysis (Supplementary Description, available as Supplementary data at *IJE* online). The highest educational level achieved was categorized as: (1) low—junior general secondary education or lower [International Standard Classification of Education (ISCED) level 0, 1 or 2]; (2) middle—secondary vocational education and senior general secondary education (ISCED level 3 or 4); and (3) high—higher vocational education or university (ISCED level 5 or 6).^28^ Income level was categorized as: (1) <1000 euro/month; (2) 1000–2000 euro/month; (3) 2000–3000 euro/month; and (4) >3000 euro/month. Welfare and unemployment status were both binary variables obtained from questions ‘I am on national assistance benefit’ and ‘I am unemployed/looking for a job’, respectively. The ISED score was subsequently categorized into quartiles, with higher quartiles indicating more disadvantaged individuals.

### Lifestyle risk index and demographics

Six lifestyle factors (i.e. smoking status, alcohol consumption, diet quality, physical activity, TV-watching time and sleep time) were selected to form the lifestyle risk index. Smoking status was categorized into never, former and current smoker. Alcohol intake and dietary consumption were derived from an externally validated 110-item semi-quantitative food-frequency questionnaire (FFQ) that assessed food consumption over the past month.^29^ Heavy drinking was defined as >40 or >20 g/day alcohol consumption for men and women, respectively.^30^ The Lifelines Diet Score (LLDS) was calculated to assess the overall diet quality based on the FFQ. This score ranks the relative intake of nine food groups with positive health effects (vegetables, fruit, whole-grain products, legumes/nuts, fish, oils/soft margarines, unsweetened dairy, coffee and tea) and three food groups with negative health effects (red/processed meat, butter/hard margarines and sugar-sweetened beverages). The development of this score is described in detail elsewhere.^31^ Non-occupational moderate-to-vigorous physical activity (MVPA) was calculated in minutes per week from the validated Short QUestionnaire to ASsess Health-enhancing physical activity (SQUASH) data, which incorporated leisure-time and commuting physical activities, including sports, at moderate [4.0–6.4 metabolic equivalent of task (MET)] to vigorous (≥6.5 MET) intensity.^32^ TV-watching time and sleep time were recorded in hours per day.

The lifestyle risk index was based on former publications from the ‘45 and Up Study’ cohort^23^ and the ‘UK Biobank’ cohort;^25^ and each lifestyle factor was categorized into a dichotomized variable (point 0 indicated healthy and point 1 indicated unhealthy). Participants were assigned one point for each unhealthy lifestyle factor (current smoker, heavy drinker, lowest two quintiles of LLDS, <75 min/week of vigorous physical activity or <150 min/week of moderate physical activity or less than the equivalent combination of MVPA, ≥4 h/day of TV-watching; <7 or >9 h of sleep time per day). Points were summed to create an unweighted index ranging from 0 to 6 for each participant, for which a higher index indicated an unhealthier lifestyle. In sensitivity analyses, the lifestyle risk index was further classified into three categories: participants who scored 0 or 1 were classified as the least unhealthy lifestyle; and those who scored 2 or 3 were classified as a moderately unhealthy lifestyle; and those who scored 4, 5 or 6 were classified as the most unhealthy lifestyle (Supplementary Table S1, available as Supplementary data at *IJE* online). Body mass index (BMI) was calculated by dividing the weight in kilograms by the square of the height in metres.

### Statistical analysis

Nominal variables are presented as frequencies [*n*, (%)] or percentage (%). Continuous variables were shown as mean ± standard deviation or median [interquartile range (IQR)].

We analysed the associations of ISED and NSED with the lifestyle risk index using linear mixed-effect models. Each neighbourhood was treated as a single unit in our study [the median number of participants per neighbourhood was 101 (IQR: 39–213)] and their corresponding neighbourhood code was treated as a random intercept in all linear mixed-effect models. First, we investigated the associations of ISED or NSED in relation to the lifestyle risk index (0–6, ordinal variable). ISED and NSED were first entered into the model separately (Model 1) and then combined and adjusted for potential confounders (Model 2 – Model 1 plus age and sex; Model 3 – Model 2 plus BMI). Second, we investigated whether NSED modified the association between ISED and the lifestyle risk index (Model 4). Interactions between ISED and NSED on the lifestyle risk index were tested by treating ISED and NSED as continuous variables, and by fitting an interaction term between the two variables (i.e. ISED by NSED). We further stratified our analyses with participants in the least socio-economically disadvantaged quartile who resided in the least socio-economically disadvantaged neighbourhoods as the reference group. When an interaction was observed, additional linear-regression analyses were performed stratified by NSED and ISED, respectively.

Sensitivity analyses included models with single individual-level measures of SED (education or income). Additional sensitivity analyses included treating the lifestyle risk index as a categorical variable and using those six single lifestyle factors from the lifestyle risk index as the outcome, respectively. Sensitivity analysis with additional adjustment for neighbourhood-level education (percentage of participants with low education) collected from the Lifelines cohort was also conducted because the neighbourhood-level education information was unavailable in the CBS Neighbourhood Statistics. All statistical analyses were conducted using Stata, version 13.1 (StataCorp, Texas, USA) or RStudio version 3.5.2 (RStudio, PBC, Boston, USA).

## Results

Of the 77 244 participants included in this study, 49 879 (64.6%) had the least unhealthy lifestyle (0 or 1 unhealthy lifestyle factor), 24 604 (31.9%) had a moderately unhealthy lifestyle (2 or 3 unhealthy lifestyle factors), whereas only 2760 (3.6%) had the most unhealthy lifestyle (4, 5 or 6 unhealthy lifestyle factors) (Supplementary Table S1, available as Supplementary data at *IJE* online). With increasing ISED quartiles, participants were more likely to have a higher lifestyle risk index (Supplementary Figure S2, available as Supplementary data at *IJE* online), have a higher BMI, be female and be older (Table 1). Moreover, the least socio-economically disadvantaged individuals were more likely to reside in the least disadvantaged neighbourhoods (Table 1), although the correlation coefficient was weak between ISED and NSED (*r* = 0.19, *P* < 0.001, Supplementary Table S2, available as Supplementary data at *IJE* online).

**Table 1:** Characteristics of individuals at different individual socio-economic disadvantage (ISED) levels

		ISED			
	Total	Q1 (least disadvantaged)	Q2	Q3	Q4 (most disadvantaged)
	77 244	*n* = 19 854	*n* = 145 01	*n* = 198 80	*n* = 23 009
Sex, male (%)	41.4	48.0	40.7	38.4	38.7
Age (years)	46 (40–53)	44 (38–51)	46 (40–51)	45 (39–50)	49 (43–58)
NSED [*n* (%)]					
Q1 (least disadvantaged)	16 358 (21.2)	6299 (31.7)	3539 (24.4)	3463 (17.4)	3057 (13.3)
Q2	15 829 (20.5)	4271 (21.5)	3238 (22.3)	4068 (20.5)	4252 (18.4)
Q3	15 773 (20.4)	3582 (18.1)	2799 (19.3)	4473 (22.5)	4919 (21.4)
Q4	15 298 (19.8)	3095 (15.6)	2697 (18.6)	4217 (21.2)	5289 (23.0)
Q5 (most disadvantaged)	13 986(18.1)	2607 (13.1)	2228 (15.4)	3659 (18.4)	5492 (23.9)
BMI (kg/m^2^)	26.4 ± 4.3	25.5 ± 3.8	26.1 ± 4.0	26.5 ± 4.4	27.1 ± 4.6
Underweight	381 (0.5)	101 (0.5)	53 (0.4)	96 (0.5)	131 (0.6)
Normal weight	31 570 (40.9)	9813 (49.4)	6055 (41.8)	7960 (40.0)	7742 (33.7)
Overweight	32 104 (41.6)	7751 (39.1)	6258 (43.1)	8178 (41.1)	9917 (43.1)
Obese	13 163(17.0)	2182 (11.0)	2129 (14.7)	3645 (18.4)	5207 (22.6)
Education level (%)					
High	30.1	99.9	17.1	0	4.1
Middle	39.3	0	59.2	99.6	8.5
Low	30.3	0	23.0	0	87.3
Income [euro/month (%)]					
<1000	3.5	0	0	0	11.6
1000–2000	19.7	0	17.1	27.9	31.3
2000–3000	30.1	27.7	0.3	50.7	33.2
>3000	32.0	62.7	82.2	0	1.6
Welfare [yes (%)]	1.2	0	0	0	4.2
Unemployment [yes (%)]	3.6	0	0	0	12.2
**Lifestyle behaviours**					
LLDS	24.3 ± 6.0	25.4 ± 5.7	24.4 ± 5.9	23.9 ± 5.8	23.8 ± 6.1
Alcohol (g/day)	7.2 ± 9.0	7.8 ± 8.4	7.8 ± 9.0	6.5 ± 8.5	7.0 ± 9.8
Heavy drinker [*n* (%)]	2032 (2.6)	432 (2.2)	437 (3.0)	439 (2.2)	724 (3.2)
TV-watching time (hours/day)	2.5 ± 1.3	2.1 ± 1.0	2.5 ± 1.1	2.6 ± 1.2	3.1 ± 1.4
≥4 [*n* (%)]	14 347 (18.6)	1496 (7.5)	2054 (14.1)	3503 (17.6)	7294 (31.7)
Sleep time (hours/day)	7.4 ± 0.9	7.4 ± 0.8	7.4 ± 0.8	7.4 ± 0.9	7.5 ± 1.0
<7 or >9 [*n* (%)]	12 463 (16.1)	2954 (14.9)	2266 (15.63)	3063 (15.4)	4180 (18.1)
Smoking status [*n* (%)]					
Current	15 516 (20.1)	2522 (12.7)	2935 (20.2)	4104 (20.6)	5955 (25.9)
Former	27 584 (35.7)	6611 (33.3)	5295 (36.5)	6780 (34.1)	8898 (38.7)
Never	34 144 (44.2)	10 721 (54.0)	6271 (43.3)	8996 (45.3)	8156 (35.4)
MVPA (min/week)	180 (60-360)	210 (90-360)	180 (60-360)	180 (60-360)	180 (60-360)
Below recommendation [*n* (%)]	20 674 (26.8)	4185 (21.1)	3767 (26.0)	5637 (28.4)	7085 (30.8)
Lifestyle risk index [*n* (%)]					
0 best	22 807 (29.5)	7983(40.2)	4549 (31.4)	5471 (27.5)	4804 (20.9)
1	27 072 (35.1)	7283(36.7)	5097 (35.1)	7145 (36.0)	7547 (32.8)
2	17 091 (22.1)	3346 (16.9)	3115 (21.5)	4613 (23.2)	6017 (26.2)
3	7513 (9.7)	1002 (5.0)	1313 (9.1)	1970 (9.9)	3228 (14.0)
4	2338(3.0)	208 (1.0)	366 (2.5)	596 (3.0)	1168 (5.1)
5	396 (0.5)	32(0.2)	60 (0.4)	80 (0.4)	224 (1.0)
6 worst	27(0.03)	0	1 (0.03)	5 (0.03)	21 (0.09)

* ISED, individual socio-economic disadvantage; NSED, neighbourhood socio-economic disadvantage; BMI, body mass index; LLDS, Lifelines Diet Score; MVPA, non-occupational moderate-to-vigorous physical activity. Missing data: education (0.3%), income (14.7%).

**Figure 1:**
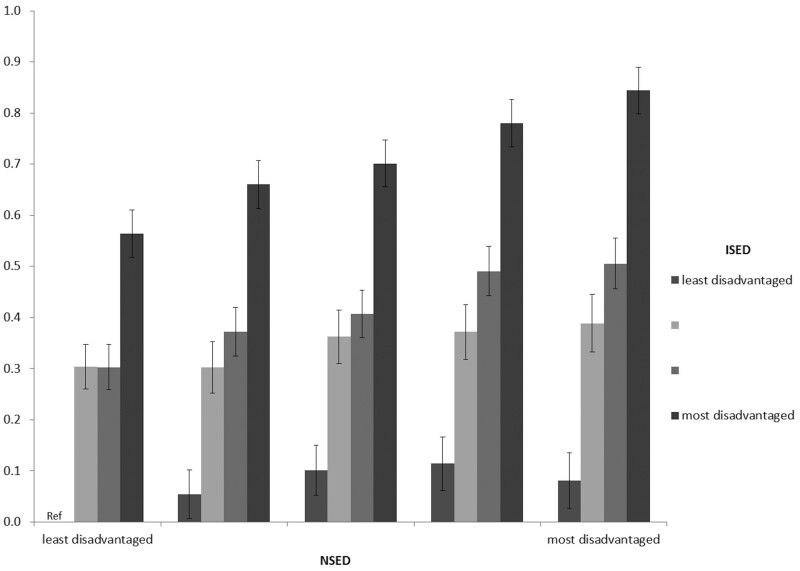
Mixed-model coefficient of the joint association of individual socio-economic disadvantage (ISED) and neighbourhood socio-economic disadvantage (NSED) with the lifestyle risk index, adjusted for age, sex and body mass index (BMI) (reference group: least socio-economically disadvantaged individuals and neighbourhoods); random effect of neighbourhood estimate (beta-coefficient 0.008, 95% CI: 0.006–0.011); intraclass correlation coefficient (ICC) 0.008 (95% CI: 0.006–0.10).

Multilevel modelling results are shown in Table 2. ISED [Q4 vs Q1: 0.58, 95% confidence interval (CI): 0.56–0.60, *P* < 0.001] and NSED [Q5 vs Q1: 0.32, 95% CI: 0.28–0.36, *P* < 0.001] were positively associated with the lifestyle risk index (Model 1, Table 2). According to the linear mixed-effect models, the magnitude of the associations (beta-coefficient) for participants who were in Q4 and Q2 of ISED were 0.64 (95% CI: 0.62–0.66, *P* < 0.001) and 0.27 (95% CI: 0.25–0.30, *P* < 0.001) compared with the reference Q1 ISED group, respectively. A positive interaction was found between NSED and ISED on the lifestyle risk index (beta-coefficient 0.016, 95% CI: 0.011–0.021, *P*_interaction_ < 0.001) (Table 2); and the association between ISED and the lifestyle risk index was steeper for those who resided in a more disadvantaged neighbourhood (Figure 1). Because of the positive interaction between ISED and NSED, analyses were repeated and stratified by ISED quartiles (Table 3). The results showed that the strength of the adjusted associations between ISED and the lifestyle risk index were highest at the most disadvantaged neighbourhood quintile (Q5). In this quintile of NSED (Q5), the estimated beta-coefficient was 0.81 (95% CI: 0.76–0.87, *P* < 0.001, Model 2) for those who were the most individually socio-economically disadvantaged, which was higher compared with individuals who were less individually socio-economically disadvantaged. In the least disadvantaged neighbourhoods, the association magnitude was 0.58 (95% CI: 0.54–0.63, *P* < 0.001, Model 2) higher for participants who were in the highest ISED quartile (Table 3), compared with those in the lowest ISED quartile. Additional adjustment for BMI (Model 3) only slightly attenuated the associations at all ISED or NSED levels. When treating participants in the lowest ISED quartile as well as the lowest NSED quintile as the reference group, the likelihood of having a higher lifestyle risk index was higher across all NSED levels among participants who were the most individually socio-economically disadvantaged, compared with those who were the least socio-economically disadvantaged (Figure 1). Furthermore, the gradient of the association across ISED levels (Q4 vs Q1) was larger for participants who resided in the most disadvantaged neighbourhood compared with those residing in the least disadvantaged neighbourhood (Figure 1 and Supplementary Figure S3, available as Supplementary data at *IJE* online).

**Table 2:** Independent associations of individual socio-economic disadvantage (ISED) and neighbourhood socio-economic disadvantage (NSED) with lifestyle risk index

	Model 1		Model 2		Model 3		Model 4	
	beta (95% CI)	*P*-trend	beta (95% CI)	*P*-trend	beta (95% CI)	*P*-trend	beta (95% CI)	*P*-value
ISED								
Q4 (most disadvantaged)	0.58 (0.56–0.60)	<0.001	0.66 (0.64–0.68)	<0.001	0.64 (0.62–0.66)	<0.001		
Q3	0.32 (0.30–0.35)		0.35 (0.33–0.38)		0.34 (0.32–0.36)			
Q2	0.25 (0.23–0.27)		0.28 (0.26–0.30)		0.27 (0.25–0.30)			
Q1 (least disadvantaged)	Ref		ref		ref			
Random effect, estimate	0.018 (0.015–0.022)							
ICC	0.015 (0.013–0.019)							
NSED								
Q5 (most disadvantaged)	0.32 (0.28–0.36)	<0.001	0.18 (0.15–0.22)	<0.001	0.17 (0.14–0.21)	<0.001		
Q4	0.28 (0.24–0.33)		0.16 (0.12–0.20)		0.15 (0.11–0.18)			
Q3	0.20 (0.16–0.24)		0.10 (0.07–0.14)		0.09 (0.06–0.13)			
Q2	0.13 (0.09–0.17)		0.06 (0.02–0.09)		0.05 (0.02–0.08)			
Q1 (least disadvantaged)	Ref		ref		ref			
Random effect, estimate	0.020 (0.016–0.024)		0.010 (0.0079–0.013)		0.009 (0.007–0.011)		0.008 (0.007–0.11)	
ICC	0.016 (0.013–0.020)		0.0089 (0.0070–0.011)		0.0077 (0.0059–0.010)		0.0076 (0.0058–0.0099)	
Interaction: ISED^*^NSED							0.016 (0.011–0.021)	<0.001

* Model 1: ISED or NSED; Model 2: ISED and NSED+ age+ sex; Model 3: Model 2+ body mass index (BMI); Model 4: Model 3+ ISED*NSED; ICC, intraclass correlation coefficient.

**Table 3:** Associations between individual socio-economic disadvantage (ISED) and the lifestyle risk index stratified by neighbourhood socio-economic disadvantage (NSED**)**

	Model 1		Model 2		Model 3	
	beta (95% CI)	*P*-trend	beta (95% CI)	*P*-trend	beta (95% CI)	*P*-trend
NSED Q5 (most disadvantaged)						
Q4 (most disadvantaged)	0.72 (0.67–0.77)	<0.001	0.81 (0.76–0.87)	<0.001	0.79 (0.74–0.85)	<0.001
Q3	0.42 (0.37–0.48)		0.46 (0.40–0.51)		0.44 (0.39–0.50)	
Q2	0.30 (0.23–0.36)		0.33 (0.27–0.39)		0.32 (0.26–0.38)	
Q1 (least disadvantaged)	ref		Ref		ref	
NSED Q4						
Q4 (most disadvantaged)	0.62 (0.57–0.67)	<0.001	0.71 (0.66–0.76)	<0.001	0.68 (0.64–0.73)	<0.001
Q3	0.37 (0.32–0.42)		0.41 (0.35–0.46)		0.39 (0.34–0.44)	
Q2	0.25 (0.19–0.31)		0.28 (0.22–0.33)		0.27 (0.21–0.32)	
Q1 (least disadvantaged)	ref		Ref		ref	
NSED Q3						
Q4 (most disadvantaged)	0.55 (0.51–0.60)	<0.001	0.65 (0.60–0.70)	<0.001	0.61 (0.57–0.66)	<0.001
Q3	0.30 (0.26–0.35)		0.33 (0.28–0.38)		0.31 (0.26–0.36)	
Q2	0.25 (0.20–0.31)		0.28 (0.23–0.33)		0.26 (0.21–0.32)	
Q1 (least disadvantaged)	ref		Ref		ref	
NSED Q2						
Q4 (most disadvantaged)	0.55 (0.51–0.59)	<0.001	0.64 (0.60–0.69)	<0.001	0.62 (0.57–0.66)	<0.001
Q3	0.32 (0.27–0.36)		0.35 (0.31–0.39)		0.33 (0.29–0.38)	
Q2	0.24 (0.19–0.29)		0.27 (0.22–0.32)		0.26 (0.21–0.21)	
Q1 (least disadvantaged)	ref		Ref		ref	
NSED Q1 (least disadvantaged)						
Q4 (most disadvantaged)	0.50 (0.46–0.55)	<0.001	0.58 (0.54–0.63)	<0.001	0.55 (0.50–0.59)	<0.001
Q3	0.29 (0.25–0.33)		0.33 (0.28–0.37)		0.30 (0.26–0.34)	
Q2	0.28 (0.24–0.32)		0.31 (0.27–0.36)		0.30 (0.26–0.34)	
Q1 (least disadvantaged)	ref		Ref		ref	

* Model 1: ISED; Model 2: Model 1+ age+ sex; Model 3: Model 2+ body mass index (BMI).

Supplementary analyses have shown that the relative risk ratio to be in the most unhealthy lifestyle category (i.e. lifestyle risk index higher than 3) among the participants from the highest quartile of ISED was 8.23 (95% CI: 7.13–9.49, *P* < 0.001) times higher than those in the lowest ISED quartile (Supplementary Table S3, available as Supplementary data at *IJE* online). The neighbourhood-disadvantage level was also positively associated with lifestyle risk index categories, with participants residing in the most disadvantaged neighbourhoods having a 1.84 (95% CI: 1.62–2.10, *P* < 0.001) times higher relative risk ratio of being in the most unhealthy lifestyle category compared with those who lived in the least disadvantaged neighbourhoods (Supplementary Table S3, available as Supplementary data at *IJE* online). Because of the positive interaction between ISED and NSED, analyses were repeated and stratified by NSED quintiles (Supplementary Table S4, available as Supplementary data at *IJE* online). The magnitude of the adjusted association between NSED and the lifestyle risk index was the highest among participants who were the most individually socio-economically disadvantaged (Supplementary Table S4, available as Supplementary data at *IJE* online). Sensitivity analyses using only education or income as an indicator for ISED (Supplementary Tables S5 andS6, available as Supplementary data at *IJE* online) as well as categorizing the lifestyle risk index into three classes as the outcome (Supplementary Table S3, available as Supplementary data at *IJE* online) showed the same pattern as our main results. Individuals who had the lowest income or education and resided in the most disadvantaged neighbourhoods had the highest likelihood of having a higher lifestyle risk index. Moreover, the patterns of interactions between NSED and education or income were also similar to the patterns between NSED and ISED (Supplementary Tables S5 andS6, available as Supplementary data at *IJE* online). However, some variations were shown for alcohol intake and MVPA, when each lifestyle factor was tested separately in the same model (Supplementary Tables S7 andS8, available as Supplementary data at *IJE* online).

## Discussion

In this large population-based study, we found that both ISED and NSED were positively associated with the lifestyle risk index. More importantly, the association between ISED and the lifestyle risk index was positively modified by NSED. Subgroup analyses revealed that the gradient of the association between ISED and the lifestyle risk index was steeper for those living in the most disadvantaged neighbourhoods.

To our knowledge, the current study is the first to simultaneously investigate the relationship of ISED, NSED and their interactive effects with an index of a broad range of lifestyle risk factors. Our study extends previous knowledge by demonstrating that the higher vulnerability of practising an unhealthy lifestyle for individuals residing in socio-economically disadvantaged neighbourhoods applies to a wider range of lifestyle factors than previously understood, including both traditional and emerging lifestyle factors such as TV-watching and sleep time. Our findings are partly consistent with previous studies showing that NSED was associated with a higher chance of having more unhealthy lifestyle factors net of ISED.^15^ The only systematic review of 22 studies found that a higher level of NSED was consistently associated with smoking and physical inactivity independently of ISED, whereas evidence of fruit/vegetable intake and excessive alcohol consumption was ambiguous.^15^ In the present study, we focused on a composite lifestyle risk index, rather than studying a single lifestyle factor. There are two major considerations for that. First, previous evidence suggests that lifestyle risk factors tended to cluster in different patterns within the population.^23^ Studying the effects of NSED on a single lifestyle factor could lead to inaccurate estimates, as their coexisting lifestyle risk factors are not simultaneously accounted for. Second, single lifestyle risk factor cannot fully capture one’s overall lifestyle risk profile, as those lifestyle factors were found to have synergistic risk contributions to one’s health outcomes.^23–25^

The underpinning mechanisms of the steeper gradient associations between ISED and the lifestyle risk index across NSED strata may be explained by several socio-health theoretical models, i.e. the double-jeopardy model, fundamental-cause theory and collective-resources model.^20^^,^^22^^,^^33^ In general, those three models all emphasize that individuals who are more socio-economically disadvantaged will be particularly worse off if they live in a disadvantaged neighbourhoods, because (i) they originally have fewer individual health resources and (ii) living in a neighbourhood with fewer health resources is expected to exacerbate one’s health more if one is already disadvantaged compared with their less disadvantaged neighbours. On the contrary, individuals who are less disadvantaged will be less affected by neighbourhood disadvantage, as they are always able to get access to health resources and depend less on their residing neighbourhoods. From another point of view, in addition to unfavourable resources, previous evidence also suggests that the neighbourhood may serve as a social platform for the spread of certain health beliefs and social norms.^34^^,^^35^ For those facing an unfavourable social environment as well as limited resources, individuals who are less disadvantaged may be more resilient and resistant to such negative factors because of their higher level of self-perceived control and knowledge for avoiding such unhealthy lifestyle behaviours.^36^^,^^37^

Our findings of the steeper gradient association between ISED and the unhealthy lifestyle risk index for individuals living in disadvantaged neighbourhoods provide two important public health implications. First, while conducting lifestyle interventions with a focus on addressing individual-level socio-economic inequalities, it is of equal importance to consider the socio-economic inequalities originating from the living neighbourhood, particularly with additional support for those who are of low individual socio-economic status. As the basic single census unit, the neighbourhood also provides a geographically tangible platform for conducting such public health interventions, which thus may help to improve the reach of health programmes for those vulnerable groups.^38^ Second, given the concrete evidence that lifestyle factors are the most important modifiable behavioural risk factors for the prevention of non-communicable diseases,^39^ public health initiatives directed towards disadvantaged neighbourhoods, in terms of both physical and social resources, may have the potential to achieve substantial public health benefits and ameliorate the persistent health inequalities within society.^40^

The strengths of this study include the relatively immobile physical and social environment of the study population, thus limiting the potential influences of the fast-changing environment and population mobility on an individual’s lifestyle factors. In fact, we only observed ∼10% of the total participants who moved between 2011 (baseline) and 2016 (second follow-up). Furthermore, our study is the first to thoroughly investigate the extent to which NSED modified the association between ISED and a spectrum of unhealthy lifestyle factors. We also conducted numerous sensitivity analyses supporting the robustness of our findings. Nevertheless, there are also limitations. First, neighbourhood-level education data were not available from the CBS Neighbourhood Statistics used for the construction of NSED. Thus, our estimated neighbourhood effects might have missed the potential influences of neighbourhood educational level. However, sensitivity analysis with additional adjustment for neighbourhood-level education (percentage of participants with low education) did not materially change the results (Supplementary Table S9, available as Supplementary data at *IJE* online). Second, there might be some misclassifications of the unweighted lifestyle risk index in more disadvantaged groups because of social desirability bias. Thus, the proportion of individuals with high lifestyle risk index might be underestimated, although the distribution of the lifestyle risk index is comparable to that in a previous study.^25^ In fact, misclassification of lifestyle in a more disadvantaged group would flatten the association between ISED and the lifestyle risk index, indicating that the associations would be even more pronounced with an accurate classification. In addition, we were not able to provide more detailed information about smoking status such as the period of cessation and the number of cigarettes, because the quality of the data in this part of the questionnaire was unfortunately insufficient due to missing data. Third, the Lifelines cohort is a single cohort study from a region with a predominantly Caucasian population (>99%) in the Netherlands—a country with a well-developed social-security system. This may limit its generalizability to populations of other ethnicity and in a different social context. Fourth, participants with missing lifestyle factors (25.4%) and NSED (13.7%) were excluded from the current study, which could possibly introduce selection bias. However, the characteristics of excluded participants did not differ substantially from those of the study population; still, participants with missing lifestyle or NSED data were more likely to report low or missing income data (Supplementary Table S10, available as Supplementary data at *IJE* online). Finally, no causal inferences should be drawn from our findings given the cross-sectional nature of our study, although additional adjustment for BMI may to some extent help to reduce the potential bias caused by reverse causation, as individuals with high BMI might alter their lifestyle factors before the entry of the study.

In conclusion, this study illustrates that NSED, in addition to ISED, was associated with a higher likelihood of practising an unhealthy lifestyle. More importantly, the association between ISED and the lifestyle risk index was positively modified by NSED. In other words, the gradient of the association between ISED and the lifestyle risk index was steeper for individuals living in the most disadvantaged neighbourhoods. These findings suggest that public health initiatives addressing lifestyle-related socio-economic health differences should not only target individual lifestyles, but also consider neighbourhood factors, in particular providing more health resources and social opportunities for those socio-economically disadvantaged neighbourhoods.

## Supplementary data

Supplementary data are available at *IJE* online.

## Ethics approval

The study was conducted in accordance with the Declaration of Helsinki and the protocol was approved by the Medical ethical committee of the University Medical Centre Groningen Institutional Review Board, The Netherlands (2007/152).

## Funding

This project has received funding from the European Union’s Horizon 2020 research and innovation programme under the Marie Skłodowska-Curie grant agreement No 754425. The Lifelines Biobank initiative has been made possible by funds from FES (Fonds Economische Structuurversterking), SNN (Samenwerkingsverband Noord Nederland) and REP (Ruimtelijk Economisch Programma).

## Data availability

The authors do not have the authority to share the data that support the findings of this study due to Lifelines data-access permissions, but any researchers can apply to use Lifelines data, including the variables used in this investigation. Information about access to Lifelines data is given on their website: https://www.lifelines.nl/researcher/how-to-apply.

## Supplementary Material

dyab079_Supplementary_Data
