# Supplementary material for: Separate and combined effects of individual and neighbourhood socio-economic disadvantage on health-related lifestyle risk factors: a multilevel analysis
Source: Int J Epidemiol. 2021 Apr 24;50(6):1959–69. doi: 10.1093/ije/dyab079 (PMC8743118; doi:10.1093/ije/dyab079)
Supplement: dyab079_Supplementary_Data [file dyab079_supplementary_data.docx]

**Supplementary Table S1.** Distribution of the lifestyle risk index

| Lifestyle risk index | Index classes | Frequency (n) | Frequency (class, n) | Percentage (%) | Percentage (class, %) |
| --- | --- | --- | --- | --- | --- |
| 0 | least unhealthy | 22807 | 49879 | 29.53 | 64.58 |
| 1 |  | 27072 |  | 35.05 |  |
| 2 | moderate unhealthy | 17091 | 24604 | 22.13 | 31.86 |
| 3 |  | 7513 |  | 9.73 |  |
| 4 | most unhealthy | 2338 | 2761 | 3.03 | 3.57 |
| 5 |  | 396 |  | 0.51 |  |
| 6 |  | 27 |  | 0.03 |  |

**Supplementary Table S2.** Spearman Correlation coefficients between all independent variables

| Correlation | Lifestyle risk index | ISED | NSED | BMI | Age | Gender |
| --- | --- | --- | --- | --- | --- | --- |
| Lifestyle risk index | 1 |  |  |  |  |  |
| ISED | 0.21 | 1 |  |  |  |  |
| p | <0.001 |  |  |  |  |  |
| NSED | 0.10 | 0.19 | 1 |  |  |  |
| p | <0.001 | <0.001 |  |  |  |  |
| BMI | 0.12 | 0.14 | 0.06 | 1 |  |  |
| p | <0.001 | <0.001 | <0.001 |  |  |  |
| Age | -0.06 | 0.17 | -0.03 | 0.12 | 1 |  |
| p | <0.001 | <0.001 | <0.001 | <0.001 |  |  |
| Gender | -0.12 | 0.07 | 0.01 | -0.12 | -0.01 | 1 |
| p | <0.001 | <0.001 | 0.005 | <0.001 | 0.01 |  |

*ISED: individual socio-economic disadvantage, NSED: neighbourhood socio-economic disadvantage, BMI: body mass index.

**Supplementary Table S3.** Independent multinomial logistical regressions of individual socio-economic disadvantage (ISED) and neighbourhood socio-economic disadvantage (NSED) with categorised lifestyle risk index (reference: least unhealthy category)

|  | Most unhealthy | | Moderate unhealthy | | Least unhealthy | |
| --- | --- | --- | --- | --- | --- | --- |
|  | rrr (95%CI) | p | rrr (95%CI) | p |  |  |
| ISED |  |  |  |  | Reference | |
| Q4 (most disadvantaged) | 8.23 (7.13-9.49) | <0.001 | 2.81 (2.69-2.95) | <0.001 |  |  |
| Q3 | 3.47 (2.98-4.03) |  | 1.82 (1.74-1.91) |  |  |  |
| Q2 | 2.96 (2.52-3.48) |  | 1.65 (1.57-1.73) |  |  |  |
| Q1 (least disadvantaged) | 1 (ref) |  | 1 (ref) |  |  |  |
| NSED |  |  |  |  |  |  |
| Q5 (most disadvantaged) | 1.84 (1.62-2.10) | <0.001 | 1.29 (1.23-1.36) | <0.001 |  |  |
| Q4 | 1.56 (1.36-1.78) |  | 1.26 (1.20-1.33) |  |  |  |
| Q3 | 1.36 (1.19-1.56) |  | 1.17 (1.11-1.23) |  |  |  |
| Q2 | 1.06 (0.92-1.22) |  | 1.12 (1.06-1.17) |  |  |  |
| Q1 (least disadvantaged) | 1 (ref) |  | 1 (ref) |  |  |  |
| Interaction: ISED*NSED | 1.03 (1.00-1.06) | 0.02 | 1.01 (1.00-1.02) | 0.03 |  |  |

* Adjusted for age, sex, and body mass index (BMI); lifestyle risk index >=4: most unhealthy, 2-3: moderate healthy; 0-1: least unhealthy; rrr: relative risk ratio.

**Supplementary Table S4.** Individual socio-economic disadvantage (ISED)-level specific associations of neighbourhood socio-economic disadvantage (NSED) and the lifestyle risk index

|  | Model 1 | | Model 2 | | Model 3 | |
| --- | --- | --- | --- | --- | --- | --- |
|  | beta (95%CI) | p trend | beta (95%CI) | p trend | beta (95%CI) | p trend |
| ISED Q4(most disadvantaged) |  |  |  |  |  |  |
| Q5 (most disadvantaged) | 0.32 (0.27-0.37) | <0.001 | 0.29 (0.24-0.34) | <0.001 | 0.28 (0.23-0.33) | <0.001 |
| Q4 | 0.24 (0.19-0.29) |  | 0.21 (0.16-0.26) |  | 0.20 (0.15-0.25) |  |
| Q3 | 0.16 (0.11-0.21) |  | 0.13 (0.08-0.18) |  | 0.13 (0.07-0.18) |  |
| Q2 | 0.09 (0.04-0.14) |  | 0.09 (0.03-0.14) |  | 0.08 (0.03-0.14) |  |
| Q1 (least disadvantaged) | ref |  | ref |  | ref |  |
| Q3 |  |  |  |  |  |  |
| Q5 (most disadvantaged) | 0.24 (0.19-0.29) | <0.001 | 0.22 (0.17-0.27) | <0.001 | 0.21 (0.16-0.26) | <0.001 |
| Q4 | 0.21 (0.16-0.25) |  | 0.19 (0.14-0.24) |  | 0.18 (0.13-0.23) |  |
| Q3 | 0.13 (0.08-0.17) |  | 0.10 (0.06-0.15) |  | 0.10 (0.05-0.15) |  |
| Q2 | 0.07 (0.02-0.12) |  | 0.06 (0.01-0.11) |  | 0.06 (0.01-0.11) |  |
| Q1 (least disadvantaged) | ref |  | ref |  | ref |  |
| Q2 |  |  |  |  |  |  |
| Q5 (most disadvantaged) | 0.12 (0.07-0.18) | <0.001 | 0.10 (0.05-0.16) | <0.001 | 0.09 (0.04-0.15) | <0.001 |
| Q4 | 0.09 (0.04-0.15) |  | 0.07 (0.02-0.12) |  | 0.06 (0.005-0.11） |  |
| Q3 | 0.08 (0.03-0.14) |  | 0.06 (0.01-0.12) |  | 0.05 (0.002-0.11) |  |
| Q2 | 0.0003 (-0.05-0.05) |  | -0,005 (-0.006-0.05) |  | -0.01 (-0.06-0.04) |  |
| Q1 (least disadvantaged) | ref |  | ref |  | ref |  |
| ISED Q1 (least disadvantaged) |  |  |  |  |  |  |
| Q5 (most disadvantaged) | 0.11 (0.06-0.15) | <0.001 | 0.10 (0.06-0.14) | <0.001 | 0.09 (0.05-0.13) | <0.001 |
| Q4 | 0.12 (0.08-0.16) |  | 0.12 (0.08-0.16) |  | 0.10 (0.06-0.14) |  |
| Q3 | 0.11 (0.07-0.15) |  | 0.11 (0.07-0.15) |  | 0.10 (0.06-0.13) |  |
| Q2 | 0.04 (0.005-0.08) |  | 0.04 (0.007-0.08) |  | 0.03 (-0.003-0.07) |  |
| Q1 (least disadvantaged) | ref |  | ref |  | ref |  |

*Model 1: NSED; Model 2: model1+age+sex; Model 3: model2+ body mass index (BMI).

**Supplementary Table S5.** Independent linear mixed effect regressions of education level and neighbourhood socio-economic disadvantage (NSED) with the lifestyle risk index

|  | Model 1 | | Model 2 | | Model 3 | | Model 4 | |
| --- | --- | --- | --- | --- | --- | --- | --- | --- |
|  | beta (95%CI) | p trend | beta (95%CI) | p trend | beta (95%CI) | p trend | beta (95%CI) | p |
| Education |  |  |  |  |  |  |  |  |
| Low | 0.56 (0.54-0.58) | <0.001 | 0.64 (0.62-0.66) | <0.001 | 0.62 (0.60-0.64) | <0.001 |  |  |
| Medium | 0.30 (0.28-0.32) |  | 0.28 (0.26-0.30) |  | 0.30 (0.29-0.32) |  |  |  |
| High | ref |  | ref |  | ref | |  |  |
| Random effect, estimate | 0.018 (0.016-0.023) |  |  |  |  |  |  |  |
| ICC | 0.016 (0.013-0.019) |  |  |  |  |  |  |  |
| NSED |  |  |  |  |  |  |  |  |
| Q5 (most disadvantaged) | 0.32 (0.28-0.36) | <0.001 | 0.21 (0.18-0.25) | <0.001 | 0.20 (0.17-0.24) | <0.001 |  |  |
| Q4 | 0.28 (0.24-0.33) |  | 0.16 (0.13-0.20) |  | 0.15 (0.12-0.19) |  |  |  |
| Q3 | 0.20 (0.16-0.24) |  | 0.11 (0.07-0.14) |  | 0.10 (0.07-0.13) |  |  |  |
| Q2 | 0.13 (0.09-0.17) |  | 0.06 (0.03-0.10) |  | 0.06 (0.02-0.09) |  |  |  |
| Q1 (least disadvantaged) | ref |  | ref |  | ref | |  |  |
| Random effect, estimate | 0.020 (0.016-0.024) |  | 0.008 (0.006-0.011) |  | 0.008 (0.006-0.011) |  | 0.008 (0.006-0.011) |  |
| ICC | 0.016 (0.013-0.020) |  | 0.009 (0.007-0.012) |  | 0.007 (0.006-0.009) |  | 0.007 (0.005-0.010) |  |
| Interaction: Education*NSED | / |  | / |  | / |  | 0.020 (0.013-0.027) | <0.001 |

*Model 1: Education or NSED; Model 2: Education and NSED+ age+ sex; Model 3: model2+ body mass index (BMI); Model 4: Model3+Education*NSED; ICC: Intraclass Correlation Coefficient.

**Supplementary Table S6.** Independent linear mixed effect regressions of income level and neighbourhood socio-economic disadvantage (NSED) with the lifestyle risk index

|  | Model 1 | | Model 2 | | Model 3 | | Model 4 | |
| --- | --- | --- | --- | --- | --- | --- | --- | --- |
|  | beta (95%CI) | p trend | beta (95%CI) | p trend | beta (95%CI) | p trend | beta (95%CI) | p |
| Income |  |  |  |  |  |  |  |  |
| Low | 0.52 (0.48-0.56) | <0.001 | 0.61 (0.56-0.65) | <0.001 | 0.59 (0.54-0.63) | <0.001 |  |  |
| Medium-lower | 0.34 (0.32-0.36) |  | 0.37 (0.35-0.39) |  | 0.35 (0.33-0.38) |  |  |  |
| Medium-higher | 0.17 (0.15-0.19) |  | 0.18 (0.16-020) |  | 0.17 (0.15-0.19) |  |  |  |
| High | ref |  | ref |  | ref |  |  |  |
| Random effect, estimate | 0.024 (0.020-0.028) |  |  |  |  |  |  |  |
| ICC | 0.020 (0.017-0.024) |  |  |  |  |  |  |  |
| NSED |  |  |  |  |  |  |  |  |
| Q5 (most disadvantaged) | 0.32 (0.28-0.36) | <0.001 | 0.21 (0.17-0.25) | <0.001 | 0.19 (0.15-0.23) | <0.001 |  |  |
| Q4 | 0.28 (0.24-0.33) |  | 0.21 (0.17-0.25) |  | 0.19 (0.15-0.23) |  |  |  |
| Q3 | 0.20 (0.16-0.24) |  | 0.15 (0.11-0.19) |  | 0.13 (0.10-0.17) |  |  |  |
| Q2 | 0.13 (0.09-0.17) |  | 0.09 (0.05-0.13) |  | 0.08 (0.04-0.12) |  |  |  |
| Q1 (least disadvantaged) | ref |  | ref |  | ref |  |  |  |
| Random effect, estimate | 0.020 (0.016-0.024) |  | 0.016 (0.013-0.02) |  | 0.013 (0.010-0.016) |  | 0.013 (0.011-0.017) |  |
| ICC | 0.016 (0.013-0.020) |  | 0.014 (0.011-0.017) |  | 0.011 (0.009-0.014) |  | 0.012 (0.009-0.015) |  |
| Interaction: Income*NSED | / |  | / |  | / |  | 0.011 (0.005-0.018) | 0.001 |

*Model 1: Income or NSED; Model 2: Income and NSED+ age+ sex; Model 3: model2+ body mass index (BMI); Model 4:Model3+ Income*NSED; ICC: Intraclass Correlation Coefficient.

**Supplementary Table S7.** Independent generalized linear mixed regressions of individual socio-economic disadvantage (ISED) and neighbourhood socio-economic disadvantage (NSED) with single lifestyle risk score from the lifestyle risk index

| Single lifestyle factor score | Smoking | | TV | | Sleep | | LLDS | | Alcohol | | MVPA | |
| --- | --- | --- | --- | --- | --- | --- | --- | --- | --- | --- | --- | --- |
|  | OR (95%CI) | p | OR (95%CI) | p | OR (95%CI) | p | OR (95%CI) | p | OR (95%CI) | p | OR (95%CI) | p |
| ISED |  |  |  |  |  |  |  |  |  |  |  |  |
| Q4 (most disadvantaged) | 2.85 (2.70-3.00) | <0.001 | 4.39 (4.13-4.68) | <0.001 | 1.19 (1.12-1.25） | <0.001 | 2.15 (2.06-2.25) | <0.001 | 1.28 (1.13-1.46) | <0.001 | 1.56 (1.49-1.64) | <0.001 |
| Q3 | 1.84 (1.74-1.95) | <0.001 | 2.40 (2.25-2.57) | <0.001 | 1.04 (0.98-1.10) | 0.2 | 1.66 (1.59-1.73) | <0.001 | 1.01 (0.88-1.16) | 0.8 | 1.34 (1.27-1.40) | <0.001 |
| Q2 | 1.84 (1.74-1.95) | <0.001 | 1.88 (1.75-2.02) | <0.001 | 1.06 (0.99-1.13) | 0.05 | 1.45 (1.38-1.52) | <0.001 | 1.38 (1.20-1.58) | <0.001 | 1.25 (1.19-1.32) | <0.001 |
| Q1 (least disadvantaged) | 1 (ref) |  | 1 (ref) |  | 1 (ref) |  | 1 (ref) |  | 1 (ref) |  | 1 (ref) |  |
| NSED |  |  |  |  |  |  |  |  |  |  |  |  |
| Q5 (most disadvantaged) | 1.51 (1.40-1.63) | <0.001 | 1.55 (1.42-1.69) | <0.001 | 1.19 (1.11-1.28) | <0.001 | 1.07 (0.99-1.14) | 0.05 | 1.08 (0.91-1.29) | 0.4 | 0.96 (0.87-1.07) | 0.4 |
| Q4 | 1.29 (1.19-1.39) | <0.001 | 1.23 (1.13-1.35) | <0.001 | 1.06 (0.99-1.14) | 0.1 | 1.09 (1.02-1.16) | 0.01 | 0.99 (0.83-1.18) | 0.9 | 1.35 (1.21-1.50) | <0.001 |
| Q3 | 1.15 (1.07-1.24) | <0.001 | 1.06 (0.97-1.16) | 0.2 | 1.00 (0.93-1.07) | 0.9 | 1.07 (1.01-1.14) | 0.03 | 0.89 (0.74-1.06) | 0.2 | 1.34 (1.22-1.48） | <0.001 |
| Q2 | 1.05 (0.97-1.13) | 0.2 | 1.03 (0.94-1.12) | 0.5 | 0.96 (0.89-1.03) | 0.2 | 1.06 (0.99-1.13) | 0.06 | 0.98 (0.82-1.16) | 0.8 | 1.23 (1.12-1.36） | <0.001 |
| Q1 (least disadvantaged) | 1 (ref) |  | 1 (ref) |  | 1 (ref) |  | 1 (ref) |  | 1 (ref) |  | 1 (ref) |  |
| Interaction: ISED*NSED | 1.02 (1.01-1.03) | 0.005 | 1.02 (1.00-1.03) | 0.007 | 1.03 (1.02-1.05) | <0.001 | 1.00 (0.99-1.01) | 0.6 | 0.99 (0.96-1.02) | 0.4 | 0.99 (0.99-1.00) | 0.6 |

*LLDS: Lifelines Diet Score, MVPA: non-occupational moderate-to-vigorous physical activity; Interaction obtained separately as the main model by treating ISES and NSES as continuous variables; all adjusted for age, sex, and body mass index (BMI).

**Supplementary Table S8.** Independent linear mixed regressions of individual socio-economic disadvantage (ISED) and neighbourhood socio-economic disadvantage (NSED) with Lifelines Diet Score (LLDS), alcohol consumption, moderate-to-vigorous physical activity (MVPA), and lifestyle score2 (excluding alcohol and MVPA)

|  | Diet | | Alcohol | | MVPA | | Score 2 | |
| --- | --- | --- | --- | --- | --- | --- | --- | --- |
|  | beta (95%CI) | p | beta (95%CI) | p | beta (95%CI) | p | beta (95%CI) | p |
| ISED |  |  |  |  |  |  |  |  |
| Q4 (most disadvantaged) | -2.39 (-2.50;-2.28) | <0.001 | -0.39 (-0.56;-0.22) | <0.001 | -3.69 (-9.43;2.05) | 0.2 | 0.54 (0.53-0.56) | <0.001 |
| Q3 | -1.60 (-1.71;-1.49) | <0.001 | -0.61 (-0.78;-0.44) | <0.001 | -5.22 (-10.96;0.52) | 0.08 | 0.28 (0.26-0.30) | <0.001 |
| Q2 | -1.14 (-1.27;-1.03) | <0.001 | 0.48 (0.30-0.67) | <0.001 | -1.29 (-7.44;4.87) | 0.7 | 0.22 (0.20-0.24) | <0.001 |
| Q1 (least disadvantaged) | ref |  | ref |  | ref |  | ref |  |
| NSED |  |  |  |  |  |  |  |  |
| Q5 (most disadvantaged) | -0.22 (-0.40;-0.03) | 0.02 | 0.0008 (-0.33;0.33) | 0.9 | 1.88 (-8.70;12.46) | 0.7 | 0.17 (0.14-0.20) | <0.001 |
| Q4 | -0.31 (-0.50;-0.12) | 0.001 | -0.08 (-0.40;0.30) | 0.7 | -29.99 (-40.88;-19.10) | <0.001 | 0.09 (0.06-0.12) | <0.001 |
| Q3 | -0.32 (-0.50;-0.14) | <0.001 | 0.026 (-0.29;0.34) | 0.9 | -31.63 (-41.84;-21.42) | <0.001 | 0.04 (0.01-0.07) | 0.002 |
| Q2 | -0.29 (-0.47;-0.11) | 0.002 | 0.22 (-0.10;0.54) | 0.2 | -22.01 (-32.23;-11.79) | <0.001 | 0.01 (-0.01;0.04) | 0.3 |
| Q1 (least disadvantaged) | ref |  | ref |  | ref |  | ref |  |
| Random effect | 0.28 (0.22-0.36) |  | 1.19 (0.98-1.44) |  | 1135.5 (928.3-1388.9) |  | 0.004 (0.003-0.006) |  |
| Interaction: ISED*NSED | 0.009 (-0.02;0.03) | 0.5 | -0.04 (-0.07;0.003) | 0.07 | 0.49 (-0.79;1.77) | 0.4 | 0.017 (0.013-0.020) | <0.001 |

*Diet, alcohol and MVPA were continuously variables representing LLDS, daily alcohol consumption (g) and weekly MVPA (minutes); Interaction was obtained separately from the main model by treating ISED and NSED as continuous variables; models all adjusted for age, sex and body mass index (BMI).

|  | Model 1 | | Model 2 | | |  |
| --- | --- | --- | --- | --- | --- | --- |
|  | beta (95%CI) | p trend | beta (95%CI) | | p |  |
| ISED |  |  |  | |  |  |
| Q4 (most disadvantaged) | 0.63 (0.61-0.65) | <0.001 |  | |  |  |
| Q3 | 0.33 (0.31-0.35) |  |  |  |  |  |
| Q2 | 0.27 (0.25-0.29) |  |  |  |  |  |
| Q1 (least disadvantaged) | ref |  |  |  |  |  |
| NSED |  |  |  | |  |  |
| Q5 (most disadvantaged) | 0.12 (0.08-0.15) | <0.001 |  | |  |  |
| Q4 | 0.09 (0.05-0.12) |  |  |  |  |  |
| Q3 | 0.05 (0.02-0.08) |  |  |  |  |  |
| Q2 | 0.02 (-0.01-0.05) |  |  |  |  |  |
| Q1 (least disadvantaged) | ref |  |  |  |  |  |
| Random effect, estimate | 0.006 (0.005-0.009) |  | 0.007 (0.005-0.009) | |  |  |
| ICC | 0.0058 (0.0043-0.0079) |  | 0.0059 (0.0044-0.0080) | |  |  |
| Interaction: ISED*NSED |  |  | 0.016 (0.011-0.020) |  | <0.001 |  |

**Supplementary Table S9.**  Independent linear logistical regressions of individual socio-economic disadvantage (ISED) and neighbourhood socio-economic disadvantage (NSED) with the lifestyle risk index.

*Model 1: NSED+ ISED+ age+ sex+ body mass index (BMI)+ percentage of people with low education at neighbourhood level; Model 2: model1+NSED*ISED.

**Supplementary Table S10.** Characteristics of step-wise excluded participants and study participants.

|  | Exclusion step 1 | Exclusion step 2 | Exclusion step 3 |  |
| --- | --- | --- | --- | --- |
|  | Missing lifestyle factors | Missing NEED data | age | Study population |
|  | n=38795 | n=21014 | n=15675 | n=77244 |
| Sex, male,% | 43.7 | 40.3 | 38.5 | 41.4 |
| Age, years | 43 (34-50) | 46 (38-56) | 26 (23-29) | 46 (40-53) |
| BMI, kg/m2 | 26.1 ± 4.5 | 26.0 ± 4.2 | 24.7 ± 4.2 | 26.4 ± 4.3 |
| Underweight | 0.9 | 0.7 | 2.1 | 0.5 |
| Normal weight | 44.2 | 44.8 | 57.6 | 40.9 |
| Overweight | 38.9 | 39.6 | 30.2 | 41.6 |
| Obese | 16.0 | 15.0 | 10.1 | 17.0 |
| Education level, % |  |  |  |  |
| High | 27.2 | 32.3 | 32.2 | 30.1 |
| Middle | 38.9 | 39.1 | 46.0 | 39.3 |
| Low | 33.4 | 28.5 | 21.3 | 30.3 |
| Income, euro/month, % |  |  |  |  |
| <1000 | 7.4 | 6.5 | 23.5 | 3.5 |
| 1000-2000 | 18.9 | 17.9 | 27.8 | 19.7 |
| 2000-3000 | 22.3 | 27.0 | 22.0 | 30.1 |
| >3000 | 22.0 | 32.3 | 14.4 | 32.0 |
| missing | 29.4 | 16.3 | 12.3 | 14.7 |
| Welfare, yes, % | 1.6 | 0.7 | 1.1 | 1.2 |
| Unemployment, yes, % | 3.5 | 3.3 | 3.5 | 3.6 |

* NSED: neighbourhood socio-economic disadvantage, BMI: body mass index.

**Supplementary Figure S1.** Study flow chart. *LLDS: Lifelines Diet Score, MVPA: non-occupational moderate-to-vigorous physical activity, NSED: neighbourhood socio-economic disadvantage.

Baseline adult participants: n=152728

Participants with valid and complete lifestyle data: n=113933

Excluded participants: n=38795

Missing non-occupational MVPA or not valid: n=19281

Missing smoking: n=5682

Missing LLDS or not valid: n=23365

Missing Alcohol or not valid: n=23365

Missing TV time or >12h/day: n=4641

Missing Sleep time or >17 h/day: n=4641

Participants with age 31-69 years old: n=77244

Excluded age<=30 years old or >69 years old: n=15675

Participants with valid and complete lifestyle data and NSED data: n=92919

Excluded missing NSED data: n=21014

**Supplementary Figure S2.** Lifestyle risk index distributions within individual socio-economic disadvantage (ISED) level

**Supplementary Figure S3.** Relative risk ratio (rrr) of joint multinomial logistic regression of individual socio-economic disadvantage (ISED) and neighbourhood socio-economic disadvantage (NSED) with moderate unhealthy (a) and most unhealthy (b) lifestyle groups (least unhealthy group as reference), adjusted for age and sex.

**Supplementary Description: Neighbourhood socio-economic disadvantage (NSED) and individual socio-economic disadvantage (ISED) scores**

NSED score was derived from principal component analysis (PCA) function in Stata, version 13 (StataCorp, Texas, USA) to summarize three NSED indicators from the northern three provinces: percentage of population with highest 20% income, percentage of population with lowest 20% income and percentage of population receiving social benefits. NSED data were derived from the Neighbourhood Statistics of Statistics Netherlands (CBS) in 2011, which is in accordance with Lifelines baseline assessment. Neighbourhoods with less than 10 inhabitants were excluded. Component 1 from PCA analysis with eigenvalue 2.28 and 75.8% explained variance, was selected to form the NSED score, and its corresponding eigenvectors (factor loadings) were: percentages of high income (-0.5947); percentages of low income (0.6059); percentages on social benefits (0.5284). The ISED score was subsequently categorised into quintiles.

ISED was determined using factor analysis of mixed data (FAMD) function from the package “FactorMineR” (version 2.3) in RStudio version 3.5.2 (RStudio, PBC, Boston, USA) to summarise four ISED variables at baselines: education level, income, welfare, unemployment. Multiple imputation (package “missMDA” [version 1.17]) was applied before FAMD analysis. The first principle component with eigenvalue 1.54 and 38.4% explained variation, was retained to form the ISED score, and its corresponding eigenvectors (factor loadings) were: education level (0.4720); income (0.6096); welfare (-0.4848); unemployment (-0.4130). The ISED score was subsequently categorised into quartiles.
